# Rare Pediatric Venolymphatic Malformations: Pericardial Cyst Mimickers

**DOI:** 10.1016/j.jaccas.2026.108130

**Published:** 2026-05-07

**Authors:** Shelby E. Walcott, Joseph Burns, Juan Pablo Sandoval Jones, Neil Cambronero, David Brennen, Thomas M. Glenn, Betul Yilmaz Furtun

**Affiliations:** a Department of Pediatrics, Texas Children's Hospital, Baylor College of Medicine, Houston, Texas, USA; b Division of Pediatric Cardiology, Department of Pediatrics, Texas Children's Hospital, Baylor College of Medicine, Houston, Texas, USA; c Division of Cardiothoracic Surgery, Department of Pediatrics, Texas Children's Hospital, Baylor College of Medicine, Houston, Texas, USA

**Keywords:** cardiac tumors, mediastinal mass, pediatric cardiology, pericardial cyst

## Abstract

**Background:**

Pericardial cystic masses are rare, and exceedingly so in pediatrics. The clinical course of such masses in children is poorly described.

**Case Summary:**

We report 2 cases in 3- and 7-year-old children. Both were initially thought to represent pericardial cysts on imaging, but surgical excision and histopathology confirmed diagnoses of a venolymphatic malformation and an epicardial cyst, respectively. The first patient presented with abdominal pain, and the second patient was asymptomatic. Both recovered well after an uncomplicated surgical intervention.

**Discussion:**

A small number of patients with pericardial masses will have symptoms thought to be related to compression of adjacent organs. Thus, this family of diagnoses must be considered in patients with findings suggestive of a pericardial cyst.

**Take Home Messages:**

Fluid-filled pericardial masses in children present a broad differential diagnosis that require comprehensive evaluation. Multidisciplinary decision-making and multimodal imaging were critical to the successful diagnosis and management of these patients.

## Visual Summary

**Figure:**
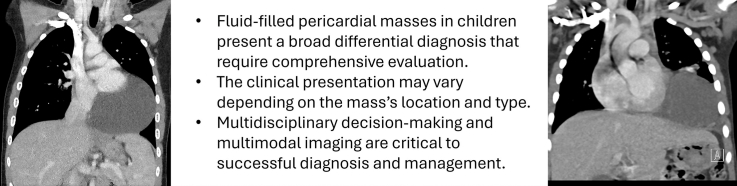

**Visual Summary:**
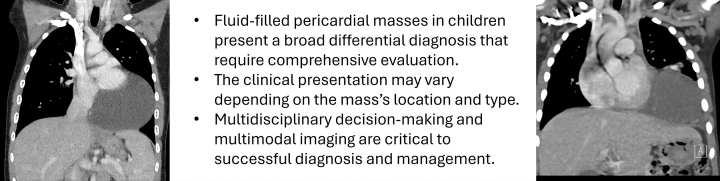
Venolymphatic Malformations Are Rare Mimickers of Pericardial Cysts in Children

Pericardial cysts are rare lesions, with an incidence of 1:100,000 in the general population.^1^, ^2^, ^3^ They are exceedingly rare in the pediatric population, with the clinical literature reporting fewer than 20 cases worldwide.^4^, ^5^, ^6^ Roughly 66% to 75% of patients are asymptomatic at the time of diagnosis, with lesions found incidentally during routine imaging.^5^^,^^6^ The remaining patients may have symptoms including chest pain or heaviness, dyspnea, and cough.^5^^,^^6^

**Take-Home Messages**

• Although rare, fluid-filled pericardial masses in children present a broad differential diagnosis that requires comprehensive evaluation.
• Similarly, although often asymptomatic, the clinical presentation may vary depending on the mass’s location and type.
• Multidisciplinary decision-making and multimodal imaging were critical to the successful diagnosis and management of the patients presented.

The differential diagnosis of pericardial cysts includes cystic masses such as congenital thoracic cysts (thyroid, thymus, esophageal, gastrogenic, bronchial, pulmonary, lymphoid, and dermoid cysts), cystic hygromas, and cystic pericardial lymphangiomas, among others.^2^ Solid tumors and metastases should also be considered.^2^

To our knowledge, there is no report in the current literature describing abdominal pain as the chief complaint for a pericardiac mass. Given the dearth of existing reports discussing pericardiac cysts in children, we report 2 unique cases of pericardial cyst mimickers. One patient was asymptomatic, and the other presented with abdominal pain.

## Case Series

### Case 1: venolymphatic malformation

A previously healthy 3-year-old boy presented to the emergency department with abdominal pain of 2 days' duration. Symptoms began with abdominal pain, nausea, decreased appetite, and decreased energy, and progressed to episodic, extreme pain with whole-body shaking and crying. On the second day of illness, he awoke with severe pain, prompting his parents to seek medical care. Two weeks prior, he had an upper respiratory infection, but symptoms resolved in a few days, and he had no respiratory symptoms since. His past medical history included a di-di twin delivery and a 7-day neonatal intensive care stay for moderate hypoxic-ischemic encephalopathy requiring therapeutic hypothermia. He followed with neurology at 4 weeks of life and required no additional treatment, imaging, or evaluation. His mother denied any long-term sequelae from his hypoxic-ischemic encephalopathy.

**Figure 1:**
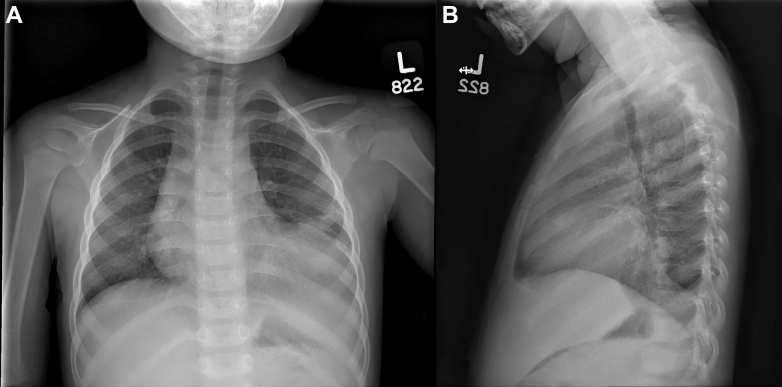
Chest X-ray Chest x-ray performed on day 1 of admission with coronal plane (A) and sagittal plane (B) showcasing a left lower lobe consolidation, addended upon review of the computed tomography scan to represent a pericardial cyst with associated streaky subsegmental atelectasis.

He was afebrile with normal vital signs. On examination, diffuse abdominal tenderness and mild guarding were noted. Laboratory results were largely unremarkable, except for a high-normal white blood cell count with neutrophilic predominance and a mildly elevated C-reactive protein. Ultrasound to evaluate intussusception was negative. Abdominal x-ray demonstrated a left basilar airspace opacification and moderate fecal burden, and chest x-ray showed a left lower lobe consolidation (Figure 1). The appendix was unable to be visualized on ultrasound, so a computed tomography (CT) scan of the abdomen and pelvis was obtained and showed a large 8.8-cm cystic mass of the left cardiophrenic recess believed to represent a pericardial cyst, with regional mass effect on the left upper and lower lobes resulting in atelectasis, as well as splenomegaly with a long axis measurement of 10.5 cm with no splenic mass.

**Figure 2:**
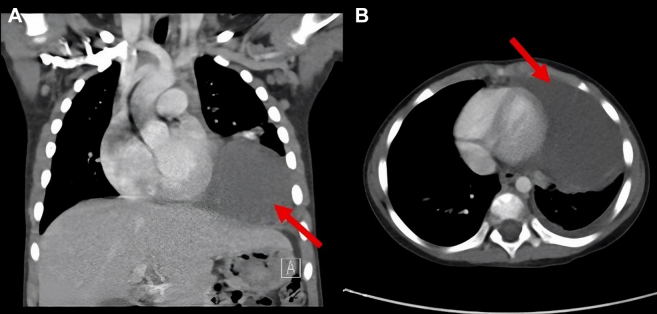
CT Chest With Contrast Computed tomography chest with contrast performed on day 3 of admission, with coronal plane (A) and axial plane (B), demonstrating a large 9.4 × 6.2 × 5.4 cm fluid density in the left cardiophrenic recess, believed to represent a pericardial cyst (red arrows).

**Figure 3:**
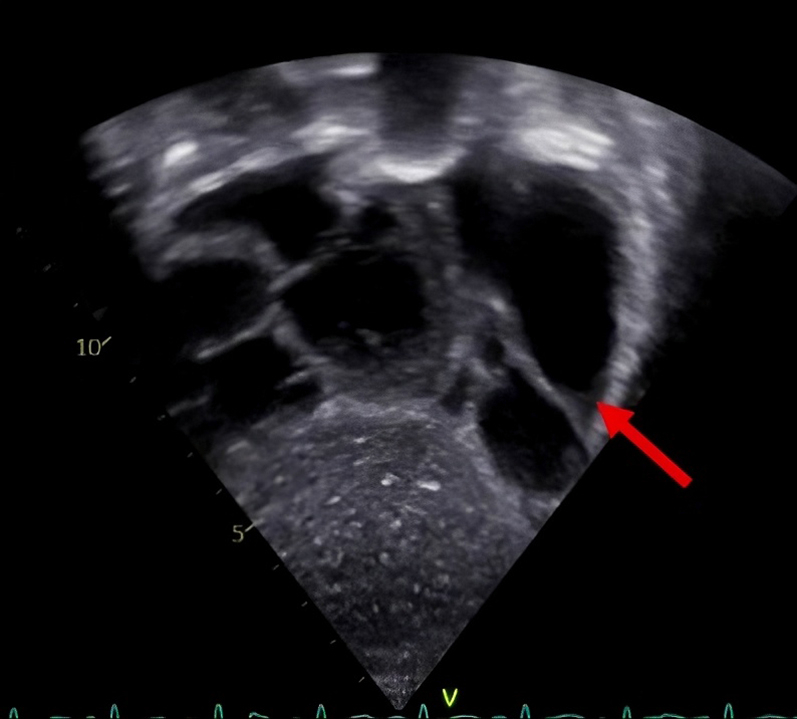
Transthoracic Echocardiogram A large cystic structure lateral to the left ventricle (red arrow) in a subcostal long-axis view.

The patient was admitted to the cardiology service. Subsequent CT chest with contrast showed a homogeneously hypoattenuating and fluid-filled mass measuring 9.4 × 6.2 × 5.4 cm in the anterior intrathoracic cavity and left cardiophrenic recess adjacent to the left heart border (Figure 2). There was mild compression of the adjacent left lateral ventricular wall as well as compression of the left lower lobe of the lung. There was also a small left pleural effusion believed to be fluid leakage from the cyst into the pleural space, vs a reactive pleural effusion. A transthoracic echocardiogram (TTE) showed a large cystic structure lateral to the left ventricle, an incidental patent foramen ovale, and normal biventricular size and systolic function (Figure 3). The electrocardiogram showed sinus tachycardia and nonspecific ST abnormality.

During his hospital stay, he remained symptomatic with significant pain and positional shortness of breath. On hospital day 5, he was presented at the Heart Center Case Conference. Surgical intervention was deferred in view of hemodynamic stability, and he was taken to the interventional catheterization laboratory for pericardiocentesis and drain placement. Seventy-five milliliters of straw-colored fluid was aspirated; the specimen was sent for infectious studies and cytology, all of which were negative. Serial TTEs showed decreased cyst size, with a moderate lateral pocket identified laterally, likely representing a smaller cyst. Infectious disease was engaged given elevated C-reactive protein and enlarged spleen, with extensive workup results negative except for a positive rhinovirus and SARS-CoV-2 viral panel, likely reflecting prior infection and unrelated to presentation. The patient was discharged on hospital day 9 with close outpatient follow-up, as the smaller cyst had been stable on serial echocardiograms and his pain had improved dramatically.

Serial outpatient echocardiograms showed a stable left loculated cystic structure. Because of a lack of resolution of the cyst and continued symptoms, the patient was deemed a candidate for elective surgical cyst removal and underwent operative resection 51 days after his initial presentation. During the operation, a tense, fluid-filled cystic mass was visualized. Fluid within the cyst was serous, and a stalk was found originating from the anterior pericardiophrenic angle against the chest wall. The cyst seemed to have grown underneath the parietal pleura and was also attached to the esophagus, diaphragm, and lung. The cyst was drained, and the fluid was sent for pathology and culture. He also underwent diagnostic esophagogastroduodenoscopy to evaluate an unusual presentation of an enteric duplication cyst arising from the distal esophagus, which revealed no mucosal defects and was reassuring against an esophageal duplication cyst. The pathology report described a 5.9 × 4.1 × 1.6 cm focally disrupted multiloculated cyst with focal adherent yellow, lobulated adipose tissue, and markers of possible venous-lymphatic malformations.

Since the operation, the patient’s abdominal pain and positional shortness of breath have resolved, appetite has returned, and activity is back to baseline. The patient is seen regularly by cardiology and most recently presented for an initial visit in the multidisciplinary Vascular Anomalies clinic, with recommendations for a 3-month postoperative CT scan to evaluate for recurrence and for genetic testing with a somatic mosaic panel assay to evaluate for somatic mosaic vascular and overgrowth syndromes.

### Case 2: epicardial cyst

**Figure 4:**
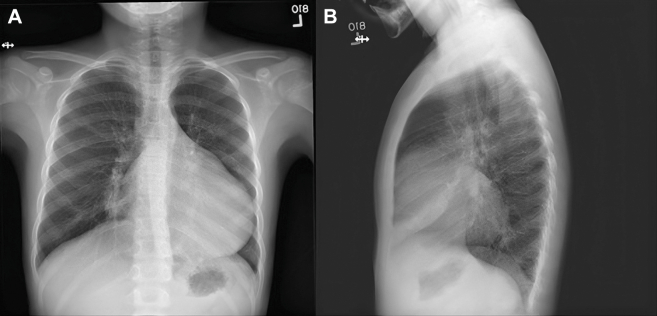
Chest X-ray Chest x-ray with coronal plane (A) and sagittal plane (B), revealing an enlarged cardiomediastinal silhouette and contours, which suggest a mediastinal abnormality.

**Figure 5:**
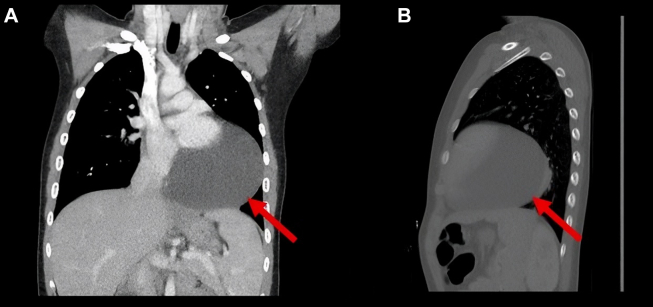
CT Chest With Contrast Computed tomography chest with contrast with coronal plane (A) and sagittal plane (B), revealing a large 8 × 7.8 × 8.9 cm pericardial cyst with mass effect on the heart (red arrows) and adjacent compressive atelectasis of the left lower lobe.

**Figure 6:**
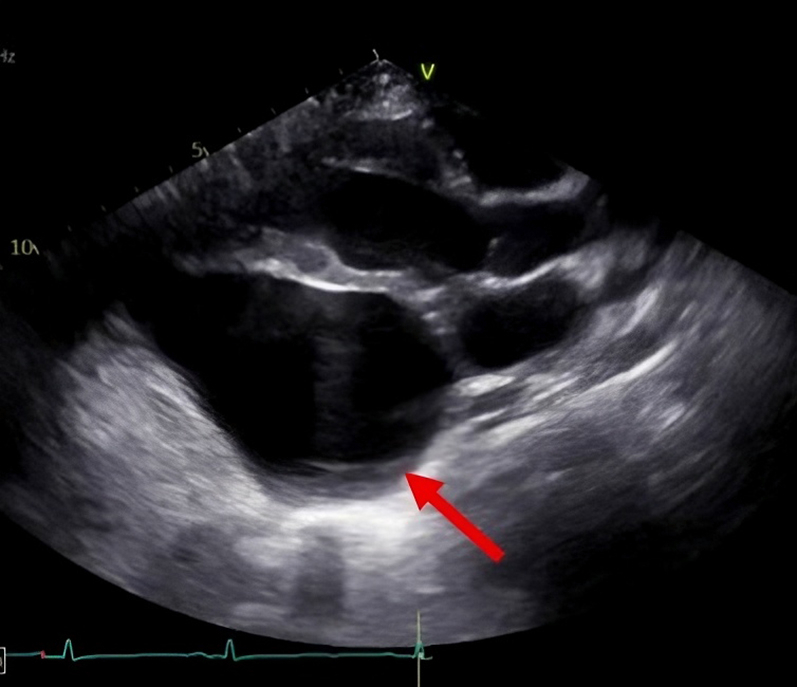
Transthoracic Echocardiogram A large cystic extracardiac mass adjacent to the left ventricular posterior wall (red arrow) in a parasternal long-axis view.

A 7-year-old boy with allergic rhinitis, atopic dermatitis, and sickle cell trait presented to the emergency department 3 separate times over the course of 1 week for unresolved fevers, headache, and cough, which progressed to left lateral chest pain during coughing episodes, abdominal pain, and post-tussive emesis. He initially presented with a 2-week history of cough and a 1-day history of fever (max 102 °F) and headache. He was diagnosed with rhinovirus and discharged with supportive care and neurology referral for his headaches. He returned a second time for continued headaches and was given valproate with resolution of his headache and discharged with education on headache hygiene. He returned 4 days later with a persistent fever (max 105 °F). His other vital signs were normal. A chest x-ray was recommended based on the history and diminished air entry on physical examination, and it revealed possible pneumonia. He was started on treatment with a 7-day course of amoxicillin. The chest x-ray also showed an enlarged cardiac silhouette, raising concern for a mediastinal abnormality (Figure 4), prompting a CT chest with IV contrast and an echocardiogram. The CT chest revealed a large 8 × 7.8 × 8.9 cm mass believed to represent a pericardial cyst with mass effect on the heart and adjacent compressive atelectasis of the left lower lobe (Figure 5). TTE demonstrated a large cystic extracardiac mass adjacent to the left ventricular posterior wall, with no evidence of tamponade physiology (Figure 6). The left ventricle demonstrated normal global systolic function with dyskinesis of the posterior/free wall, presumed to be related to the cystic structure posterior to the heart. The electrocardiogram showed a normal sinus rhythm and a nonspecific T-wave abnormality. B-natriuretic peptide and troponin were both normal. The cyst was considered an incidental finding, and he was discharged for close follow-up with cardiology.

He had no prior hospitalizations or surgeries. He had no prior known cardiac symptoms except occasional left lateral chest pain after playing soccer. Three years before presentation, he was evaluated for a heart murmur, which was felt to be an innocent murmur with no imaging performed or follow-up recommended.

His case was presented to the Heart Center 6 days after discharge, and he underwent elective surgical resection roughly 4 months after initial presentation. During the operation, the pericardium was noted to be totally intact. The cyst was entirely covered by epicardium and seemed to have originated from the myocardium of the posterior wall of the left ventricle. The cyst was carefully removed intact. The pathology report showed a 9.5 × 9 × 5.5 cm benign, mesothelial-lined cyst, consistent with an epicardial cyst. No definitive myocardial fibers were identified within the cyst wall.

Since the operation, the patient has been followed regularly by cardiology and has remained asymptomatic, with normal activity levels.

## Discussion

This case series offers a unique description of 2 children with pericardial masses initially diagnosed as pericardial cysts on preoperative imaging. Most pericardial cysts are asymptomatic and discovered incidentally. Clinical manifestations, if present, include chest pain, dyspnea, cough, or palpitations.^3^^,^^5^^,^^6^

The first patient, a 3-year-old child, presented with a chief complaint of abdominal pain and had imaging findings initially suggestive of a pericardial cyst, but were ultimately more consistent with a venolymphatic malformation. Other causes of abdominal pain were ruled out, including appendicitis, intussusception, gastroenteritis, constipation, fecal impaction, testicular torsion, pancreatitis, and urinary tract infection. A large pericardiac mass was discovered in the left cardiophrenic recess, and his abdominal pain resolved after operative resection. As such, the cause of his pain was believed to be referred pain from diaphragmatic compression of the phrenic nerve due to either the mass itself or fluid leaking into the pleural space. To our knowledge, there is no report in the current literature describing abdominal pain as the chief complaint for a pericardiac mass. This unique presentation may prompt future recognition and diagnosis of pericardiac mass cases.

The second patient’s mass was incidentally identified during a work-up for pneumonia and was thought to be consistent with a pericardial cyst on imaging and ultimately found to originate from the epicardium. Although both pericardial cysts and venolymphatic malformations may appear similar on initial imaging, pericardial cysts are typically well-defined, nonenhancing, simple fluid-filled structures, whereas venolymphatic malformations represent congenital vascular anomalies that often demonstrate multiloculated architecture, internal septations, fluid-fluid levels, and variable enhancement, with definitive distinction frequently requiring surgical and pathologic confirmation.^7^

Treatment options for pericardial cysts include observation, percutaneous drainage, surgical excision, or video-assisted thoracoscopic surgery. In asymptomatic cysts, conservative management with serial echocardiograms is often the chosen route due to the increased likelihood of spontaneous resolution.^8^ Surgery is often considered for large, symptomatic masses or when there is concern for infection,^4^ hemorrhage, or tamponade.^5^^,^^6^ There is an ongoing debate on whether aspiration or surgical resection should be the initial treatment for pericardial cysts. In 2004, the task force on the diagnosis and management of pericardial diseases of the European Society of Cardiology recommended percutaneous aspiration and ethanol sclerosis as initial treatment of congenital and inflammatory cysts, and surgical resection or video-assisted thoracotomy as second-line treatment.^9^^,^^10^ On the other hand, surgical resection has been preferred in adults with severe symptomatic pericardial cysts, due to the higher chance of pericardial cyst recurrence with aspiration.^3^ Proponents of an initial surgical approach in children argue that initial surgical removal minimizes cyst recurrence, provides definitive histologic diagnosis, and reduces potential morbidity associated with complications of pericardial cysts.^5^

Future research is warranted to guide treatment recommendations for pericardial cysts in the pediatric population and specifically to compare percutaneous aspiration vs surgical resection or video-assisted thoracoscopic surgery as initial treatment.

## Conclusions

Pericardial cysts are extremely rare in the pediatric population, and the existing literature is limited. In this case series, we describe 2 fluid-filled pericardial masses—a venolymphatic malformation and an epicardial cyst—both initially diagnosed as pericardial cysts on preoperative imaging, with definitive characterization achieved after surgical and pathologic evaluation. This series may provide clues for the further recognition and diagnosis of pericardiac masses, as well as for the timing of intervention.

## Funding Support and Author Disclosures

The authors have reported that they have no relationships relevant to the contents of this paper to disclose.
